# Petermann-factor sensitivity limit near an exceptional point in a Brillouin ring laser gyroscope

**DOI:** 10.1038/s41467-020-15341-6

**Published:** 2020-03-31

**Authors:** Heming Wang, Yu-Hung Lai, Zhiquan Yuan, Myoung-Gyun Suh, Kerry Vahala

**Affiliations:** 1 T. J. Watson Laboratory of Applied Physics, California Institute of Technology, Pasadena, CA 91125 USA; 2 Present Address: OEwaves Inc., 465 North Halstead Street, Suite 140, Pasadena, CA 91107 USA; 3 Present Address: Physics and Informatics Laboratories, NTT Research, Inc., East Palo Alto, CA 94303 USA

**Keywords:** Optical sensors, Microresonators, Nonlinear optics, Imaging and sensing

## Abstract

Exceptional points are singularities of open systems, and among their many remarkable properties, they provide a way to enhance the responsivity of sensors. Here we show that the improved responsivity of a laser gyroscope caused by operation near an exceptional point is precisely compensated by increasing laser noise. The noise, of fundamental origin, is enhanced because the laser mode spectrum loses the oft-assumed property of orthogonality. This occurs as system eigenvectors coalesce near the exceptional point and a bi-orthogonal analysis confirms experimental observations. While the results do not preclude other possible advantages of the exceptional-point-enhanced responsivity, they do show that the fundamental sensitivity limit of the gyroscope is not improved through this form of operation. Besides being important to the physics of microcavities and non-Hermitian photonics, these results help clarify fundamental sensitivity limits in a specific class of exceptional-point sensor.

## Abstract (web summary)

Operating a laser gyroscope near an exceptional point has been shown to enhance its responsivity. However, here the authors demonstrate in theory and experiment that the enhanced responsivity is exactly compensated by increased noise that is inherent to this system near the exceptional point.

## Introduction

Non-Hermitian Hamiltonians^1,2^ describing open systems can feature singularities called exceptional points (EPs)^3–5^. EPs have been experimentally realized in several systems^6–8^ and applied to demonstrate non-reciprocal transmission^9–11^ and lasing dynamics control^12–15^. Moreover, resonant frequencies become strongly dependent on externally applied perturbations near an EP, which has given rise to the concept of EP-enhanced sensing in photonics^16–19^ and electronics^20,21^. While increased sensor responsivity has been demonstrated in several systems^22–25^, signal-to-noise performance (sensitivity) has been considered only theoretically^26–30^.

Recently, strong responsivity improvement near an EP was reported in a Brillouin ring laser gyroscope by monitoring an increase in the gyroscope scale factor (i.e., transduction factor of rotation-rate into a signal)^24^. At the same time, however, measurement of the gyroscope Allan deviation versus averaging time showed that short-term laser frequency noise also increased near the EP. This noise was random-walk in nature, suggesting a fundamental origin. Moreover, it depended upon system bias relative to the EP in such a way so as to precisely compensate for the observed EP-enhanced transduction. As a result, the gyroscope’s angular random walk, the metric used to quantify short-term rotation sensitivity, was observed to maintain a constant value (i.e., independent of gyroscope bias relative to the EP). In effect, the measurements showed that gyroscope sensitivity (i.e., weakest rotation signal measurable at a given detection bandwidth) is not improved by operation near the EP even while the gyroscope responsiveness through improved transduction (scale factor) increases.

As with all laser gyroscopes, the Brillouin ring laser gyroscope measures rotations through the Sagnac effect^31^. Clockwise (cw) and counter-clockwise (ccw) lasing waves experience opposing frequency shifts when the plane of the gyroscope rotates. By mixing the two laser fields on a detector, their difference frequency therefore reveals the rotation-induced frequency shift added onto a constant bias frequency (which is at audio rates in this case^24^). Frequency noise in the beat frequency therefore determines the measurement sensitivity. This noise has both a technical component (observable on longer time scales in the Allan deviation^24^) as well as a random walk component that, absent the EP, is known to result from fundamental linewidth broadening of the Brillouin laser waves^32,33^. Significantly, subsequent measurement of the random walk component showed that none of the parameters which normally impact its magnitude (e.g., laser power, cavity Q factor) varied near the EP, therefore suggesting that frequency noise (and linewidth) is increased by way of another mechanism.

Laser linewidth can also be broadened by the Petermann factor^34–39^. This mechanism is associated with non-orthogonality of a mode spectrum, and its connection to EPs has been considered in theoretical studies of microresonators^40,41^. However, despite continued theoretical interest^42,43^, including the development of new techniques for determination of linewidth in general laser systems^44^, the observation of Petermann linewidth broadening near exceptional points was reported only recently by the Yang group in a phonon laser system^45^, and the link between Petermann-factor-induced noise and EP sensor performance is unexplored.

Here, it is shown that mode non-orthogonality induced by the EP limits the gyroscope sensitivity via Petermann-factor linewidth broadening. Indeed, analysis and measurement confirm near-perfect cancellation of the signal transduction improvement by increasing Petermann-factor noise, so that the gyroscope’s fundamental signal-to-noise ratio (SNR) and hence sensitivity is not improved by operation near the EP. These results are further confirmed using an Adler phase locking equation approach^46,47^, which is also applied to analyze the combined effect of dissipative and conservative coupling on the system.

## Results

### Biorthogonal noise enhancement theory

**Fig. 1:**
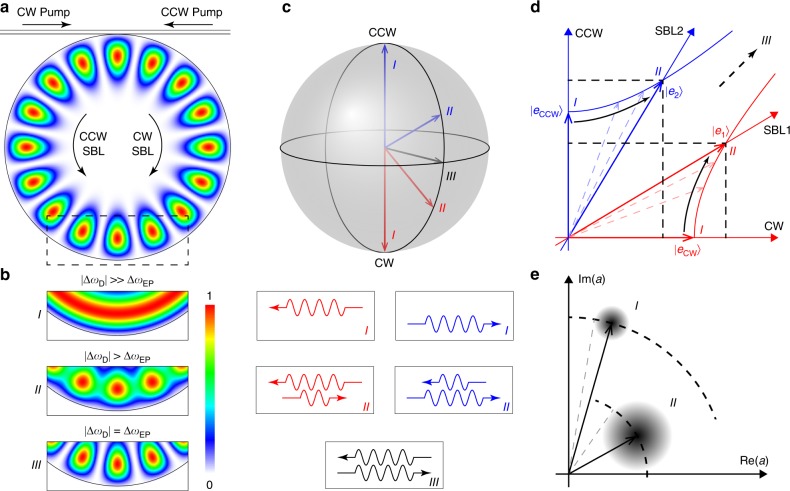
Brillouin laser linewidth enhancement near an exceptional point. **a** Diagram of whispering-gallery mode resonator with the energy distribution of an eigenmode superimposed. The eigenmode energy distribution corresponds to state III in **b**. Optical pumps on the coupling waveguide and whispering-gallery SBL modes are indicated by arrows. **b** Mode energy distributions for three different states: far from EP (state I) the eigenmodes are traveling cw or ccw waves; near EP (state II) the eigenmodes are hybrids of cw and ccw waves; at EP (state III) eigenmodes coalesce to a standing wave. **c** Bloch sphere showing the eigenstates for cases I, II and III with corresponding cw and ccw composition. **d** Illustration of the cw-ccw and SBL1-SBL2 coordinate systems. Unit vectors for states I and II are shown on each axis. As the system is steered towards the EP, the SBL axes move toward each other so that unit vectors along the SBL axes lengthen as described by the two hyperbolas. This is illustrated by decomposing a unit vector of the non-orthogonal SBL coordinate system using the orthogonal cw-ccw coordinates [e.g., (5/4, 3/4)${}^{T}$ and (3/4, 5/4)${}^{T}$ for state II]. Consequently, the field amplitude is effectively shortened in the SBL basis. **e** Phasor representation of the complex amplitude of a lasing mode for states I and II provides an interpretation of linewidth enhancement. Phasor length is shortened and noise is enhanced as the system is steered to the EP, leading to an increased phasor angle diffusion and laser linewidth enhancement (see Supplementary Note 3).

The gyroscope uses a high-Q silica whispering gallery resonator^48^ in a ring-laser configuration^32^. As illustrated in Fig. 1a, optical pumping of cw and ccw directions on the same whispering-gallery mode index induces laser action through the Brillouin process. On account of the Brillouin phase matching condition, these stimulated Brillouin laser (SBL) waves propagate in a direction opposite to their corresponding pump waves^33^. Dissipative backscattering^49^ couples the SBLs and the following Hamiltonian governs the above-laser-threshold motion^24^:

1 $$H=\left(\begin{array}{cc}{\omega }_{{\rm{cw}}}&i \Delta{\omega }_{{\rm{EP}}}/2\\ i\Delta{\omega }_{{\rm{EP}}}/2&{\omega }_{{\rm{ccw}}}\end{array}\right)$$

where $H$ describes the dynamics via $id\Psi/dt=H\Psi$ and $\Psi={\left({a}_{{\rm{cw}}},{a}_{{\rm{ccw}}}\right)}^{T}$ is the column vector of SBL mode amplitudes (square of norm is photon number). Also, $\Delta{\omega }_{{\rm{EP}}}$ is a non-Hermitian term related to the coupling rate between the two SBL modes and ${\omega }_{{\rm{cw}}}$ (${\omega }_{{\rm{ccw}}}$) is the active-cavity resonance angular frequency of the cw (ccw) SBL mode above laser threshold. The dependence of ${\omega }_{{\rm{cw}}}$, ${\omega }_{{\rm{ccw}}}$, and $\Delta{\omega }_{{\rm{EP}}}$ on other system parameters, most notably the angular rotation rate and the optical pumping frequencies, has been suppressed for clarity.

A class of EP sensors operates by measuring the frequency difference of the two system eigenmodes. This difference is readily calculated from Eq. (1) as $\Delta{\omega }_{{\rm{S}}}=\sqrt{\Delta{\omega }_{{\rm{D}}}^{2}-\Delta{\omega }_{{\rm{EP}}}^{2}}$ where $\Delta{\omega }_{{\rm{D}}}\equiv {\omega }_{{\rm{ccw}}}-{\omega }_{{\rm{cw}}}$ is the resonance frequency difference and $\Delta{\omega }_{{\rm{EP}}}$ is the critical value of $\Delta{\omega }_{{\rm{D}}}$ at which the system is biased at the EP. As illustrated in Fig. 1b, c the vector composition of the SBL modes strongly depends upon the system proximity to the EP. For $| \Delta{\omega }_{{\rm{D}}}| \gg \Delta{\omega }_{{\rm{EP}}}$ the SBL modes (unit vectors) are orthogonal cw and ccw waves. However, closer to the EP the waves become admixtures of these states that are no longer orthogonal. At the EP, the two waves coalesce to a single state vector (a standing wave in the whispering gallery). Rotation of the gyroscope in state II in Fig. 1 $\left(\left|\Delta{\omega }_{{\rm{D}}}\right|\;> \;\Delta{\omega }_{{\rm{EP}}}\right)$ introduces a perturbation to $\Delta{\omega }_{{\rm{D}}}$ whose transduction into $\Delta{\omega }_{{\rm{S}}}$ is enhanced relative to the conventional Sagnac factor^31^. This EP-induced signal-enhancement-factor (SEF) is given by^24^,

2 $${\rm{SEF}}={\left|\frac{\partial \Delta{\omega }_{{\rm{S}}}}{\partial \Delta{\omega }_{{\rm{D}}}}\right|}^{2}=\frac{\Delta{\omega }_{{\rm{D}}}^{2}}{\Delta{\omega }_{{\rm{D}}}^{2}-\Delta{\omega }_{{\rm{EP}}}^{2}}$$

where SEF refers to the signal power (not amplitude) enhancement. This factor has recently been verified in the Brillouin ring laser gyroscope^24^. The control of $\Delta{\omega }_{{\rm{D}}}$ (and in turn $\Delta{\omega }_{{\rm{S}}}$) in that work and here is possible by tuning of the optical pumping frequencies and is introduced later.

$\Delta{\omega }_{{\rm{S}}}$ is measured as the beat frequency of the SBL laser signals upon photodetection and the SNR is set by the laser linewidth. To understand the linewidth behavior a bi-orthogonal basis is used as described in Supplementary Notes 1 and 3. As shown there and illustrated in Fig. 1d, the peculiar properties of non-orthogonal systems near the EP cause the unit vectors (optical modes) to be lengthened. This lengthening results in an effectively shorter laser field amplitude. Also, noise into the mode is increased as illustrated in Fig. 1e. Because the laser linewidth can be understood to result from diffusion of the phasor in Fig. 1e, linewidth increases upon operation close to the EP. And the linewidth enhancement is given by the Petermann factor (see Supplementary Note 2),

3 $${\rm{PF}}=\frac{1}{2}\left(1+\frac{{\rm{Tr}}({H}_{0}^{{\rm{\dagger }}}{H}_{0})}{| {\rm{Tr}}({H}_{0}^{2})| }\right)=\frac{\Delta{\omega }_{{\rm{D}}}^{2}}{\Delta{\omega }_{{\rm{D}}}^{2}-\Delta{\omega }_{{\rm{EP}}}^{2}}$$

where ${\rm{Tr}}$ is the matrix trace operation and ${H}_{0}=H-{\rm{Tr}}(H)/2$ is the traceless part of $H$. As derived in Supplementary Note 2, the first part of this equation is a basis independent form and is valid for a general two-dimensional system. The second part is specific to the current SBL system. Inspection of Eqs. (2) and (3) shows that SEF = PF. As a result the SNR is not expected to improve through operation near the EP when the system is fundamental-noise limited.

### Petermann noise measurement

**Fig. 2:**
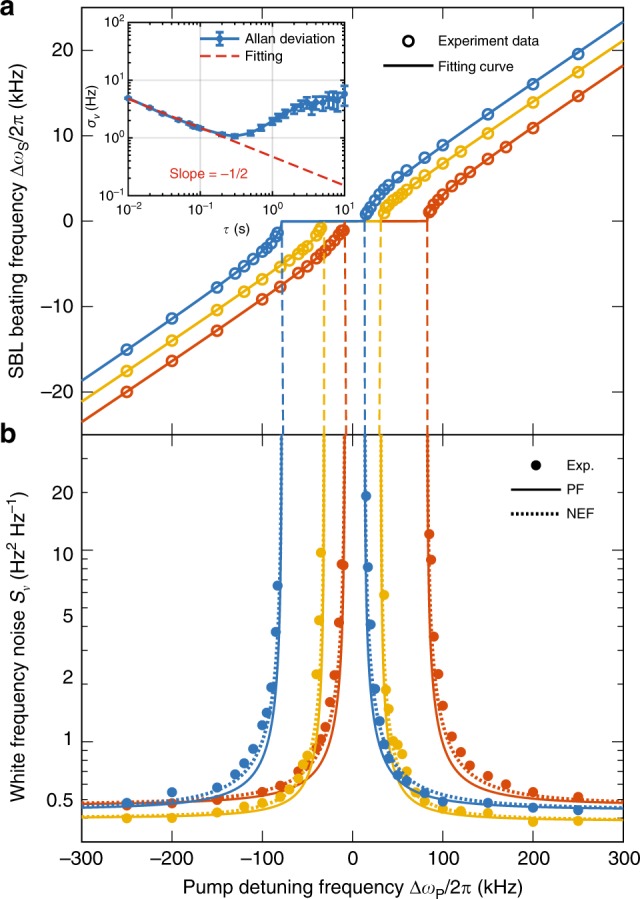
Measured linewidth enhancement of SBLs near the exceptional point. **a** Measured SBL beating frequency is plotted versus pump detuning for three distinct locking zones, corresponding SBL amplitude ratios $q$: 1.15 (blue), 1 (orange), 0.85 (red). Solid curves are theoretical fittings. Inset is a typical Allan deviation measurement of frequency (${\sigma }_{\nu }(\tau )$) versus gate time $\tau$. Error bars give the standard error of the mean. The short-term part is fitted with $\sqrt{{S}_{\nu }/(2\tau )}$ where ${S}_{\nu }$ is the one-sided power spectral density of the white frequency noise plotted in **b**. **b** Measured white frequency noise of the beating signal is determined using the Allan deviation measurement. Data point color corresponds to the amplitude ratios used in **a**. The Petermann factor PF (solid lines) and NEF (dashed lines) theoretical predictions use parameters obtained by fitting from **a**.

To verify the above predictions, the output of a single pump laser (~1553.3 nm) is divided into two branches that are coupled into cw and ccw directions of the resonator using a tapered fiber^50,51^. Both pump powers are actively stabilized. The resonator is mounted in a sealed box and a thermo-electric cooler (TEC) controls the chip temperature which is monitored using a thermistor (fluctuations are held within 5 mK). Each pumping branch has its frequency controlled using acousto-optic modulators (AOMs). SBL power is also monitored and controlled so that fluctuations are within 0.6%. Even with the control of temperature and power, the Allan deviation at longer gate times reflects technical-noise drifting that is observed to be more pronounced for operation near the EP. As described in ref. ^24^, the ccw pump laser frequency is Pound-Drever-Hall (PDH) locked to one resonator mode and the cw pump laser can then be independently tuned by the AOM. This pump detuning frequency ($\Delta{\omega }_{{\rm{P}}}$) is therefore controlled to radio-frequency precision. It is used to precisely adjust $\Delta{\omega }_{{\rm{D}}}$ and in turn $\Delta{\omega }_{{\rm{S}}}$ as shown in three sets of measurements in Fig. 2a. Here, the photodetected SBL beat frequency $\Delta{\omega }_{{\rm{S}}}$ is measured using a frequency counter. The data sets are taken for three distinct SBL output amplitude ratios as discussed further below. A solid curve fitting is also presented using $\Delta{\omega }_{{\rm{S}}}=\pm \!\sqrt{\Delta{\omega }_{{\rm{D}}}^{2}-\Delta{\omega }_{{\rm{EP}}}^{2}}$, where $\Delta{\omega }_{{\rm{D}}}=\frac{\gamma /{\rm{\Gamma }}}{1+\gamma /{\rm{\Gamma }}}\Delta{\omega }_{{\rm{P}}}+\frac{1}{1+\gamma /{\rm{\Gamma }}}\Delta{\omega }_{{\rm{Kerr}}}$ (see Supplementary Note 4). Also, $\gamma$ is the photon decay rate, ${\rm{\Gamma }}$ is the Brillouin gain bandwidth^33^, and $\Delta{\omega }_{{\rm{Kerr}}}$ is a Kerr effect correction that is explained below. As an aside, the data plot and theory show a frequency locking zone, the boundaries of which occur at the EP.

The frequency counter data are also analyzed as an Allan deviation (Adev) measurement (Fig. 2a inset). The initial roll-off of the Adev features a slope of $-1$/2 corresponding to white frequency noise^52^. This was also verified in separate measurements of the beat frequency using both an electrical spectrum analyzer and a fast Fourier transform. The slope of this region is fit to $\sqrt{{S}_{\nu }/(2\tau )}$ where ${S}_{\nu }$ is the one-sided spectral density of the white frequency noise. Adev measurement at each of the detuning points in Fig. 2a is used to infer the ${S}_{\nu }$ values that are plotted in Fig. 2b. There, a frequency noise enhancement is observed as the system is biased toward an EP. Also plotted is the Petermann factor noise enhancement (Eq. (3)). Aside from a slight discrepancy at intermediate detuning frequencies (analyzed further below), there is an overall excellent agreement between theory and measurement. The frequency noise levels measured in Fig. 2b are consistent with fundamental SBL frequency noise (see Methods). Significantly, the fundamental nature of the noise, the good agreement between the PF prediction (Eq. (3)) and measurement in Fig. 2b, and separate experimental work^24^ that has verified the theoretical form of the SEF (Eq. (2)) confirm that SEF = PF so that the fundamental sensitivity limit of the gyroscope is not improved by operation near the EP.

### Adler noise analysis

While the Petermann factor analysis provides very good agreement with the measured results, we also derived an Adler-like coupled mode equation analysis for the Brillouin laser system. This approach is distinct from the bi-orthogonal framework and, while more complicated, provides additional insights into the system behavior. Adapting analysis applied in the noise analysis of ring laser gyroscopes^47^, a noise enhancement factor NEF results (see Supplementary Note 4),

4 $${\rm{NEF}}=\frac{\Delta{\omega }_{{\rm{D}}}^{2}+\Delta{\omega }_{{\rm{EP}}}^{2}/2}{\Delta{\omega }_{{\rm{D}}}^{2}-\Delta{\omega }_{{\rm{EP}}}^{2}}$$

It is interesting that this result, despite the different physical context of the Brillouin laser system, has a similar form to one derived for polarization-mode-coupled laser systems^53^. The PF and NEF predictions are shown in Fig. 2b and the Adler-derived NEF correction provides slightly better agreement with the data at the intermediate detuning values.

### Adler locking bandwidth analysis

**Fig. 3:**
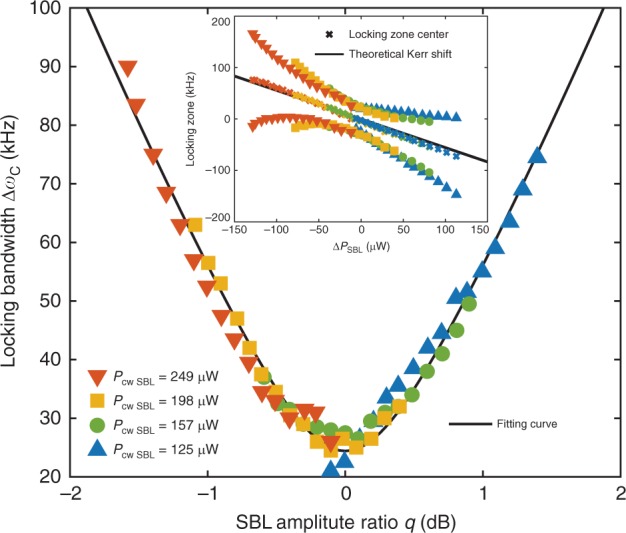
Locking zone bandwidth versus SBL amplitude ratio. Measured locking zone bandwidth is plotted versus amplitude ratio $q$ of the SBL lasers. The cw power is held constant at four values (see legend) to create the data composite. The solid black curve is Eq. (5). Inset: the measured locking zone boundaries are plotted versus the SBL power differences (${\rm{\Delta }}{P}_{{\rm{SBL}}}={P}_{{\rm{ccw}}}-{P}_{{\rm{cw}}}$). Colors and symbols correspond to the main panel. The center of the locking zone is also indicated and is shifted by the Kerr nonlinearity which varies as the SBL power difference. Black line gives the theoretical prediction (no free parameters).

The Adler approach is also useful to explain a locking zone dependence upon SBL amplitudes observed in Fig. 2a. As shown in Supplementary Note 4, this variation can be explained through the combined action of the Kerr effect and intermodal coupling coefficients of both dissipative and conservative nature. Specifically, the locking bandwidth is found to exhibit the following dependence upon the amplitude ratio $q=| {a}_{{\rm{ccw}}}/{a}_{{\rm{cw}}}|$ of the SBL lasers,

5 $$\Delta{\omega }_{{\rm{EP}}}^{2}={\left(\frac{{\rm{\Gamma }}}{{\rm{\Gamma }}+\gamma }\right)}^{2}\left[{\left(q+\frac{1}{q}\right)}^{2}| \kappa {|}^{2}+{\left(q-\frac{1}{q}\right)}^{2}| \chi {|}^{2}\right]$$

where $\kappa$ is the dissipative coupling and $\chi$ is the conservative coupling between cw and ccw SBL modes. The locking zone boundaries in terms of pump detuning frequency have been measured (Fig. 3 inset) for a series of different SBL powers. Using this data, the locking bandwidth is expressed in pump frequency detuning ($\Delta{\omega }_{{\rm{P}}}$) units using $\Delta{\omega }_{C}\equiv (1+{\rm{\Gamma }}/\gamma )\Delta{\omega }_{{\rm{EP}}}$ and plotted versus $q$ in the main panel of Fig. 3. The plot agrees well with Eq. (5) (fitting shown in black) and gives $| \kappa |$ = 0.93 kHz, $| \chi |$ = 8.21 kHz.

Finally, the center of the locking band is shifted by the Kerr effect and (in pump frequency detuning $\Delta{\omega }_{{\rm{P}}}$ units) can be expressed as $-({\rm{\Gamma }}/\gamma )\Delta{\omega }_{{\rm{Kerr}}}$, where $\Delta{\omega }_{{\rm{Kerr}}}=\eta \left({\left|{a}_{{\rm{ccw}}}\right|}^{2}-{\left|{a}_{{\rm{cw}}}\right|}^{2}\right)=(\eta \Delta{P}_{{\rm{SBL}}})/({\gamma }_{{\rm{ex}}}\hslash \omega )$ is the Kerr induced SBL resonance frequency difference, $\Delta{P}_{{\rm{SBL}}}={P}_{{\rm{ccw}}}-{P}_{{\rm{cw}}}$ is the output power difference of the SBLs, and ${\gamma }_{{\rm{ex}}}$ is the photon decay rate due to the output coupling. Also, $\eta ={n}_{2}\hslash {\omega }^{2}c/(V{n}_{0}^{2})$ is the single-photon Kerr-effect angular frequency shift with $\omega$ the SBL angular frequency, ${n}_{2}$ the Kerr-nonlinear refractive index of silica, $V$ the mode volume, ${n}_{0}$ the linear refractive index, and $c$ the speed of light in vacuum. If the white frequency noise floors in Fig. 2 are used to infer the resonator quality factor, then a Kerr nonlinearity value of 558 Hz μW^−1^ is predicted (see Methods). This value gives the line plot in the Fig. 3 inset (with no free parameters), which agrees with experiment.

## Discussion

Prior work has shown that Brillouin laser gyroscopes when operated near an EP have an improved responsivity (equivalently, an increase in the gyroscope’s scale factor for transduction of rotation rate into the Sagnac frequency shift)^24^. At the same time, these measurements have shown that the gyroscope’s sensitivity did not improve near the EP. We have verified through measurement and theory that mode non-orthogonality induced by the EP explains this latter result. Specifically, increasing mode non-orthogonality occurs when the two system eigenvectors (optical modes) begin to coalesce near the EP. This, in turn, increases laser frequency noise from an increasing Petermann factor and thereby reduces sensitivity. Curiously, these two mechanisms, the enhanced transduction and enhanced noise, feature an almost identical dependence upon the system’s proximity to the EP. In effect, the increased signal response in the gyroscope arising from the EP does not lead to an improvement in the minimum detectable signal (sensitivity).

It is interesting to note that a recent theoretical study of noise limitations in a class of non-lasing EP sensors showed no fundamental sensitivity advantage for operation near the EP^28^. Nonetheless it is still possible that other sensing modalities could benefit from operation near an EP. Moreover, open systems offer other potentially useful ways for transduction of rotation^54^. Also, the proposal of conservative nonlinear mode coupling provides a potential way to enhance the Sagnac effect^55–57^. The apparent divergence of the linewidth near the EP is an interesting feature of the current model and also one that agrees well with the data (at least in the range of the measurement). Nonetheless, constraints to this divergence set by the linewidth of the passive cavity loss are a subject of further study. More generally, the excellent control of the state space that is possible in the Brillouin system can provide a new platform for studies of the remarkable physics associated with exceptional points.

## Methods

### Linewidth and Allan deviation measurement

In experiments, frequency is measured in the time domain using a frequency counter and its Allan deviation is calculated for different averaging times (Fig. 2a). The Allan deviation ${\sigma }_{\nu }(\tau )$ for a signal frequency is defined by

6 $${\sigma }_{\nu }\left(\tau \right)\equiv \sqrt{\frac{1}{2\left(M-1\right)}\sum _{k=1}^{M-1}{\left({\bar{\nu }}_{k+1}-{\bar{\nu }}_{k}\right)}^{2}}$$

where $\tau$ is the averaging time, $M$ is the number of frequency measurements, and ${\overline{\nu }}_{k}$ is the average frequency of the signal (measured in Hz) in the time interval between $k\tau$ and $(k+1)\tau$. The Allan deviation follows a ${\tau }^{-1/2}$ dependence when the underlying frequency noise spectral density is white^52^ as occurs for laser frequency noise limited by spontaneous emission. White noise causes the lineshape of the laser to be a Lorentzian. White noise is also typically dominant in the Allan deviation plot at shorter averaging times where flicker noise and frequency drift are not yet important. This portion of the Allan deviation plot can be fit using ${\sigma }_{\nu }(\tau )=\sqrt{{S}_{\nu }/(2\tau )}$ where ${S}_{\nu }$ is the white frequency noise one-sided spectral density function. This result can be further converted to the Lorentzian full-width at half maximum (FWHM) linewidth $\Delta{\nu }_{{\rm{FWHM}}}$ (measured in Hz) using the conversion,

7 $${S}_{\nu }=2{\sigma }_{\nu }^{2}(\tau )\tau =\frac{1}{\pi }\Delta{\nu }_{{\rm{FWHM}}}$$

### Experimental parameters and data fitting

The resonator is pumped at the optical wavelength $\lambda =1553.3$ nm, which, subject to the Brillouin phase matching condition, corresponds to a phonon frequency (Stokes frequency shift) of approximately ${{\rm{\Omega }}}_{{\rm{phonon}}}/(2\pi )=10.8$ GHz. Quality factors of the SBL modes are measured using a Mach-Zehnder interferometer, and a loaded Q factor ${Q}_{{\rm{T}}}=88\times 1{0}^{6}$ and coupling Q factor ${Q}_{{\rm{ex}}}=507\times 1{0}^{6}$ are obtained.

The theoretical formula for the white frequency noise of the beat frequency far away from the EP reads,

8 $${S}_{\nu }={\left(\frac{{\rm{\Gamma }}}{\gamma +{\rm{\Gamma }}}\right)}^{2}\frac{\hslash {\omega }^{3}}{4{\pi }^{2}{Q}_{{\rm{T}}}{Q}_{{\rm{ex}}}}\left(\frac{1}{{P}_{{\rm{cw}}}}+\frac{1}{{P}_{{\rm{ccw}}}}\right)\left({n}_{{\rm{th}}}+{N}_{{\rm{th}}}+1\right)$$

which results from summing the Schawlow-Townes-like linewidths of the SBL laser waves^33^. In the expression, ${N}_{{\rm{th}}}$ and ${n}_{{\rm{th}}}$ are the thermal occupation numbers of the SBL state and phonon state, respectively. At room temperature, ${n}_{{\rm{th}}}\approx 577$ and ${N}_{{\rm{th}}}\approx 0$. For the power balanced case (orange data set in Fig. 2), *P*_cw_ = *P*_ccw_ = 215 μW and the predicted white frequency noise (Eq. (8)) is ${S}_{\nu }$ = 0.50 Hz^2^ Hz^−1^. For the blue (red) data set, ${P}_{{\rm{cw}}}$ (${P}_{{\rm{ccw}}}$) is decreased by 1.22 dB (1.46 dB) so that ${S}_{\nu }=$ 0.58 (0.60) Hz^2^ Hz^−1^ is calculated. On the other hand, the measured values for the blue, orange and red data sets in Fig. 2b (i.e., white frequency noise floors far from EP) give ${S}_{\nu }=$ 0.44, 0.39, 0.46 Hz^2^ Hz^−1^, respectively. The difference here is attributed to errors in Q measurement. For example, the experimental values of noise can be used to infer a corrected coupling Q factor ${Q}_{{\rm{ex}}}\approx 658\times 1{0}^{6}$. Using this value below yields an excellent prediction of the Kerr nonlinear coefficient which supports this belief.

The beating frequency in Fig. 2a is fit using the following relations:

9 $$\Delta{\omega }_{{\rm{S}}} = {\rm{sgn}}(\Delta{\omega }_{{\rm{D}}})\sqrt{\Delta{\omega }_{{\rm{D}}}^{2}-\Delta{\omega }_{{\rm{EP}}}^{2}}\\ \Delta{\omega }_{{\rm{D}}} = \frac{\gamma /{\rm{\Gamma }}}{1+\gamma /{\rm{\Gamma }}}\Delta{\omega }_{{\rm{P}}}+\frac{1}{1+\gamma /{\rm{\Gamma }}}\Delta{\omega }_{{\rm{Kerr}}}$$

where sgn is the sign function and $\gamma /{\rm{\Gamma }}$, $\Delta{\omega }_{{\rm{Kerr}}}$, and $\Delta{\omega }_{{\rm{EP}}}$ are fitting parameters. The fitting gives $\gamma /{\rm{\Gamma }}=0.076$ consistently, while $\Delta{\omega }_{{\rm{Kerr}}}$ and $\Delta{\omega }_{{\rm{EP}}}$ are separately adjusted in each data set. These parameters feature a power dependence that is fully explored in Fig. 3 and the related main text discussion.

The theoretical Kerr coefficient used in Fig. 3 can be calculated as follows. Assuming ${n}_{2}\approx 2.7\times 1{0}^{-20}$ ${\text{m}}^{2}/\text{W}\,$, ${n}_{0}=1.45$ for the silica material, and *V* = 10^7^ μm^3^ (obtained through finite-element simulations for the 36mm-diameter disk used here), gives $\eta /2\pi \approx 1{0}^{-5}$ Hz. Using the ${Q}_{{\rm{ex}}}$ corrected by the white frequency noise data (see discussion above), ${\gamma }_{{\rm{ex}}}/2\pi =299$ kHz so that $\Delta{\omega }_{{\rm{Kerr}}}/(2\pi \Delta{P}_{{\rm{SBL}}})\approx 42$ Hz μW^−1^. When $\gamma /{\rm{\Gamma }}=0.076$, the center shift of pump locking band is $-({\rm{\Gamma }}/\gamma )\Delta{\omega }_{{\rm{Kerr}}}$ = 558 Hz μW^−1^. This value agrees very well with experiment (Fig. 3 inset).

## Supplementary information

Supplementary Information

## Data Availability

The data that support the plots within this paper and other findings of this study are available from the corresponding author upon reasonable request.
