# Supplementary material for: Petermann-factor sensitivity limit near an exceptional point in a Brillouin ring laser gyroscope
Source: Nat Commun. 2020 Mar 31;11:1610. doi: 10.1038/s41467-020-15341-6 (PMC7109037; doi:10.1038/s41467-020-15341-6)
Supplement: Supplementary file 1 — Supplementary Information [file 41467_2020_15341_MOESM1_ESM.pdf]

Supplementary Information for:
Petermann-factor limited sensing near an exceptional point in a Brillouin ring laser gyroscope

Wang, Lai, Yuan et al.

Supplementary Note 1. NON-HERMITIAN HAMILTONIAN AND BI-ORTHOGONAL RELATIONS

Here we briefly review the framework for working with general non-Hermitian matrices. An n-dimensional matrix M has n eigenvalues $\mu_1,\ \mu_2,\ ...\ \mu_n$. For simplicity we will assume that all of the eigenvalues are distinct, i.e. $\mu_j \neq \mu_k$ if $j \neq k$. In this case M will have n right eigenvectors and n left eigenvectors associated with each μ_j:

$$M|v_j^{\mathrm{R}}\rangle = \mu_j|v_j^{\mathrm{R}}\rangle, \quad \langle v_j^{\mathrm{L}}|M = \langle v_j^{\mathrm{L}}|\mu_j \tag{1}$$

To make sense of the left eigenvectors, note that $M^\dagger|v_j^{\mathrm{L}}\rangle = \mu_j^*|v_j^{\mathrm{L}}\rangle$, thus the left eigenvector is the eigenstate as if loss is changed to gain and vice versa. Since M is in general non-Hermitian, there is no guarantee that $|v_j^{\mathrm{L}}\rangle = |v_j^{\mathrm{R}}\rangle$, and many of the decomposition results that hold in the Hermitian case will fail. However we note that,

$$\mu_j\langle v_j^{\mathrm{L}}|v_k^{\mathrm{R}}\rangle = \langle v_j^{\mathrm{L}}|M|v_k^{\mathrm{R}}\rangle = \mu_k\langle v_j^{\mathrm{L}}|v_k^{\mathrm{R}}\rangle \ \Rightarrow\ \langle v_j^{\mathrm{L}}|v_k^{\mathrm{R}}\rangle = 0,\ \forall j \neq k \tag{2}$$

Thus left and right eigenvectors associated with different eigenvalues are bi-orthogonal. We also note that the right eigenvectors are complete and form a set of basis (as M is non-degenerate and finite-dimensional), and we can decompose the identity matrix and M as follows:

$$1 = \sum_j \frac{|v_j^{\mathrm{R}}\rangle\langle v_j^{\mathrm{L}}|}{\langle v_j^{\mathrm{L}}|v_j^{\mathrm{R}}\rangle} \tag{3}$$

$$M = \sum_j \frac{|v_j^{\mathrm{R}}\rangle\langle v_j^{\mathrm{L}}|}{\langle v_j^{\mathrm{L}}|v_j^{\mathrm{R}}\rangle}\mu_j \tag{4}$$

where each term is a "projector" onto the eigenvectors. Again we note that $\langle v_j^{\mathrm{L}}|v_j^{\mathrm{R}}\rangle$ may be negative and even complex, which results in special normalizations of the vectors. For simplicity we will choose $\langle v_j^{\mathrm{L}}|v_j^{\mathrm{R}}\rangle = 1$ by rescaling the vectors and adjusting the relative phase (such vectors are sometimes said to be bi-orthonormal). With this normalization in place the above decompositions simplify further as follows:

$$1 = \sum_j |v_j^{\mathrm{R}}\rangle\langle v_j^{\mathrm{L}}| \tag{5}$$

$$M = \sum_j |v_j^{\mathrm{R}}\rangle\langle v_j^{\mathrm{L}}|\mu_j \tag{6}$$

We note that, as a result of using bi-orthonormal left and right vectors, the vectors, themselves, are not normalized, i.e. $\langle v_j^{\mathrm{L}}|v_j^{\mathrm{L}}\rangle$ and $\langle v_j^{\mathrm{R}}|v_j^{\mathrm{R}}\rangle$ need not be 1 for each j. There is one extra degree of freedom per mode for fixing the lengths, but the length normalization factors do not affect the physical observables if such factors are kept consistently through the calculations. In Supplementary Note 3 a "natural" normalization will be chosen when we give a physical meaning to these factors.

Supplementary Note 2. PETERMANN FACTOR OF A TWO-DIMENSIONAL HAMILTONIAN

Here we derive the Petermann factor of a two-dimensional Hamiltonian H. Denote the two normalized right (left) eigenvectors of H as $|\psi_1^{\mathrm{R}}\rangle$ and $|\psi_2^{\mathrm{R}}\rangle$ ($|\psi_1^{\mathrm{L}}\rangle$ and $|\psi_2^{\mathrm{L}}\rangle$). The Petermann factors of these two eigenmodes can then be expressed as [1]

$$\mathrm{PF}_1 = \langle\psi_1^{\mathrm{L}}|\psi_1^{\mathrm{L}}\rangle\langle\psi_1^{\mathrm{R}}|\psi_1^{\mathrm{R}}\rangle \tag{7}$$

$$\mathrm{PF}_2 = \langle\psi_2^{\mathrm{L}}|\psi_2^{\mathrm{L}}\rangle\langle\psi_2^{\mathrm{R}}|\psi_2^{\mathrm{R}}\rangle \tag{8}$$

We will first prove that $\mathrm{PF}_1 = \mathrm{PF}_2$, which can then be identified as the Petermann factor for the entire system. Note that $|\psi_1^{\mathrm{L}}\rangle$ and $|\psi_2^{\mathrm{R}}\rangle$ are orthogonal and span the two-dimensional space. As a result, the identity can be expressed using this set of basis vectors as follows:

$$1 = \frac{|\psi_1^{\mathrm{L}}\rangle\langle\psi_1^{\mathrm{L}}|}{\langle\psi_1^{\mathrm{L}}|\psi_1^{\mathrm{L}}\rangle} + \frac{|\psi_2^{\mathrm{R}}\rangle\langle\psi_2^{\mathrm{R}}|}{\langle\psi_2^{\mathrm{R}}|\psi_2^{\mathrm{R}}\rangle} \tag{9}$$

Now apply this expansion to $|\psi_1^{\mathrm{R}}\rangle$ and obtain

$$|\psi_1^{\mathrm{R}}\rangle = \frac{1}{\langle\psi_1^{\mathrm{L}}|\psi_1^{\mathrm{L}}\rangle}|\psi_1^{\mathrm{L}}\rangle + \frac{\langle\psi_2^{\mathrm{R}}|\psi_1^{\mathrm{R}}\rangle}{\langle\psi_2^{\mathrm{R}}|\psi_2^{\mathrm{R}}\rangle}|\psi_2^{\mathrm{R}}\rangle \tag{10}$$

where $\langle\psi_1^{\mathrm{L}}|\psi_1^{\mathrm{R}}\rangle = 1$ has been used. Left multiplication by $\langle\psi_1^{\mathrm{R}}|$ results in

$$\langle\psi_1^{\mathrm{R}}|\psi_1^{\mathrm{R}}\rangle = \frac{1}{\langle\psi_1^{\mathrm{L}}|\psi_1^{\mathrm{L}}\rangle} + \frac{\langle\psi_1^{\mathrm{R}}|\psi_2^{\mathrm{R}}\rangle\langle\psi_2^{\mathrm{R}}|\psi_1^{\mathrm{R}}\rangle}{\langle\psi_2^{\mathrm{R}}|\psi_2^{\mathrm{R}}\rangle} \tag{11}$$

Thus we obtain,

$$\frac{1}{\mathrm{PF}_1} = 1 - \frac{\langle\psi_1^{\mathrm{R}}|\psi_2^{\mathrm{R}}\rangle\langle\psi_2^{\mathrm{R}}|\psi_1^{\mathrm{R}}\rangle}{\langle\psi_1^{\mathrm{R}}|\psi_1^{\mathrm{R}}\rangle\langle\psi_2^{\mathrm{R}}|\psi_2^{\mathrm{R}}\rangle} \tag{12}$$

which is symmetric with respect to the indexes 1 and 2 and thereby completes the proof that $\mathrm{PF}_1 = \mathrm{PF}_2 \equiv \mathrm{PF}$.

Next, PF is expressed using the Hamiltonian instead of its eigenvectors. We begin by noting that the identity operator added to the Hamiltonian will not modify the eigenvectors. As a result, the trace can be removed from H without changing the value of PF:

$$H_0 \equiv H - \frac{1}{2}\mathrm{Tr}(H) \tag{13}$$

where Tr is the matrix trace and H_0 is the traceless part of H. Using the bi-orthogonal expansion, H_0 has the form,

$$H_0 = \mu(|\psi_1^{\mathrm{R}}\rangle\langle\psi_1^{\mathrm{L}}| - |\psi_2^{\mathrm{R}}\rangle\langle\psi_2^{\mathrm{L}}|) \tag{14}$$

where μ is the first eigenvalue. Consider next the quantity $\mathrm{Tr}(H_0^\dagger H_0)$:

$$\mathrm{Tr}(H_0^\dagger H_0) = |\mu|^2(\langle\psi_1^{\mathrm{L}}|\psi_1^{\mathrm{L}}\rangle\langle\psi_1^{\mathrm{R}}|\psi_1^{\mathrm{R}}\rangle + \langle\psi_2^{\mathrm{L}}|\psi_2^{\mathrm{L}}\rangle\langle\psi_2^{\mathrm{R}}|\psi_2^{\mathrm{R}}\rangle - \langle\psi_2^{\mathrm{L}}|\psi_1^{\mathrm{L}}\rangle\langle\psi_1^{\mathrm{R}}|\psi_2^{\mathrm{R}}\rangle - \langle\psi_1^{\mathrm{L}}|\psi_2^{\mathrm{L}}\rangle\langle\psi_2^{\mathrm{R}}|\psi_1^{\mathrm{R}}\rangle) \tag{15}$$

where we used the fact that $\mathrm{Tr}(|\alpha\rangle\langle\beta|) = \langle\beta|\alpha\rangle$. To simplify the expression, note that each of the first two terms equals PF. Moreover, the third term can be evaluated by expressing $|\psi_1^{\mathrm{L}}\rangle$ as a combination of right eigenvectors using Supplementary Eq. (10):

$$-\langle\psi_2^{\mathrm{L}}|\psi_1^{\mathrm{L}}\rangle\langle\psi_1^{\mathrm{R}}|\psi_2^{\mathrm{R}}\rangle = \frac{\langle\psi_1^{\mathrm{R}}|\psi_2^{\mathrm{R}}\rangle\langle\psi_2^{\mathrm{R}}|\psi_1^{\mathrm{R}}\rangle}{\langle\psi_2^{\mathrm{R}}|\psi_2^{\mathrm{R}}\rangle}\langle\psi_1^{\mathrm{L}}|\psi_1^{\mathrm{L}}\rangle = \mathrm{PF} - 1 \tag{16}$$

Similarly, the fourth term also equals $\mathrm{PF} - 1$. Thus

$$\mathrm{Tr}(H_0^\dagger H_0) = |\mu|^2(4\mathrm{PF} - 2) \tag{17}$$

Finally, to eliminate the eigenvalue μ we calculate,

$$\mathrm{Tr}(H_0^2) = \mu^2(\langle\psi_1^{\mathrm{L}}|\psi_1^{\mathrm{R}}\rangle^2 + \langle\psi_2^{\mathrm{L}}|\psi_2^{\mathrm{R}}\rangle^2) = 2\mu^2 \tag{18}$$

and the PF can be solved as

$$\mathrm{PF} = \frac{1}{2}\left(1 + \frac{\mathrm{Tr}(H_0^\dagger H_0)}{|\mathrm{Tr}(H_0^2)|}\right) \tag{19}$$

which completes the proof.

We note that while a Hermitian Hamiltonian with $H_0^\dagger = H_0$ results in $\mathrm{PF} = 1$, the converse is not always true. Consider the example of $H_0 = i\sigma_z$ where σ_z is the Pauli matrix. This would effectively describe two orthogonal modes with different gain, and direct calculation shows that $\mathrm{PF} = 1$.

Supplementary Note 3. FIELD AMPLITUDE AND NOISE IN A NON-ORTHOGONAL SYSTEM

Here we consider the physical interpretation of increased linewidth whereby the effective field amplitude decreases while the effective noise input increases as a result of non-orthogonality. This analysis considers a hypothetical laser mode that is part of the bi-orthogonal system. It skips key steps normally taken in a more rigorous laser noise analysis in order to make clearer the essential EP physics. A more complete study of the Brillouin laser system is provided in Supplementary Note 4.

The two-dimensional system is described by the column vector $|\Psi\rangle \leftrightarrow (a_{\rm cw}, a_{\rm ccw})^T$ whose components are the orthogonal field amplitudes $a_{\rm cw}$ and $a_{\rm ccw}$. The equation of motion reads $id|\Psi\rangle/dt = H|\Psi\rangle$, where H is the two-dimensional Hamiltonian. Now assume that $|\Psi\rangle = c_1|\psi_1^{\rm R}\rangle$, i.e. only the first eigenmode of the system is excited. We interpret c_1 as the phasor for the eigenmode. We see that $|c_1|^2 = \langle\Psi|\Psi\rangle/\langle\psi_1^{\rm R}|\psi_1^{\rm R}\rangle$ is reduced from the true square amplitude $\langle\Psi|\Psi\rangle$ by a factor of the length squared of the right eigenvector $\langle\psi_1^{\rm R}|\psi_1^{\rm R}\rangle$. The equation of motion for c_1 reads

$$i\frac{dc_1}{dt} = i\frac{d\langle\psi_1^{\rm L}|\Psi\rangle}{dt} = \langle\psi_1^{\rm L}|H_0|\psi_1^{\rm R}\rangle c_1 = \mu_1 c_1 \tag{20}$$

Here, we are assuming that the mode experiences both loss and saturable gain that are absorbed into the definition of the eigenvalue μ_1. To simplify the following calculations we set the real part of μ_1 to 0, since any frequency shift can be removed with an appropriate transformation to slowly varying amplitudes.

To introduce noise into the system resulting from the amplification process the equation of motion is modified as follows: $id|\Psi\rangle/dt = H_0|\Psi\rangle + |F\rangle$. Here, $|F\rangle \leftrightarrow (\overline{F}_{\rm cw}(t), \overline{F}_{\rm ccw}(t))^T$ is a column vector with fluctuating components. The noise correlation of these components is assumed to be given by,

$$\langle\overline{F}^*_{\rm cw}(t)\overline{F}_{\rm cw}(t')\rangle = \langle\overline{F}^*_{\rm ccw}(t)\overline{F}_{\rm ccw}(t')\rangle = \theta\delta(t-t') \tag{21}$$

$$\langle\overline{F}^*_{\rm cw}(t)\overline{F}_{\rm ccw}(t')\rangle = \langle\overline{F}^*_{\rm ccw}(t)\overline{F}_{\rm cw}(t')\rangle = 0 \tag{22}$$

where θ is a quantity with frequency dimensions. We note that the assumption of vanishing correlations between the fluctuations on different modes is not trivial. Even if the basis is orthogonal, the non-Hermitian nature of the Hamiltonian means that dissipative mode coupling will generally be present in the system. This will be associated with fluctuations that can induce off-diagonal elements in the correlation matrix. In the system studied here, we will show in Supplementary Note 4 that the main source of noise comes from the phonons and fluctuations due to the non-Hermitian Hamiltonian are negligible, thereby justifying the assumption made here. Taking account of the fluctuations, the equation of motion for c_1 can be modified as follows,

$$\frac{dc_1}{dt} = -|\mu_1|c_1 + \langle\psi_1^{\rm L}|F\rangle = -|\mu_1|c_1 + \overline{F}_1 \tag{23}$$

where the fluctuation term for the first eigenmode is defined as $\overline{F}_1 = \langle\psi_1^{\rm L}|F\rangle$. Its correlation reads

$$\langle\overline{F}^*_1(t)\overline{F}_1(t')\rangle = \theta\langle\psi_1^{\rm L}|\psi_1^{\rm L}\rangle\delta(t-t') \tag{24}$$

which, upon comparison to Supplementary Eq. (21), shows that the noise input to the right eigenvector field amplitude (c_1) is enhanced (relative to the noise input to either the cw or ccw fields alone) by a factor of the length squared of the left eigenvector $\langle\psi_1^{\rm L}|\psi_1^{\rm L}\rangle$.

We are interested in the phase fluctuations of c_1. Here, it is assumed that the mode is pumped to above threshold and is lasing. Under these conditions it is possible separate amplitude and phase fluctuations of the field. We rewrite $c_1 = |c_1|\exp(-i\phi_c)$ and obtain the rate of change of the phase variable as follows:

$$\frac{d\phi_c}{dt} = \frac{i}{2|c_1|}\left(\overline{F}_1 {\rm e}^{i\phi_c} - \overline{F}^*_1 {\rm e}^{-i\phi_c}\right) \tag{25}$$

which describes white frequency noise of the laser field (equivalently phase noise diffusion). The correlation can be calculated as

$$\langle\dot{\phi}_c(t)\dot{\phi}_c(t')\rangle = \frac{\theta}{2|c_1|^2}\langle\psi_1^{\rm L}|\psi_1^{\rm L}\rangle\delta(t-t') = \frac{\theta}{2\langle\Psi|\Psi\rangle}\langle\psi_1^{\rm R}|\psi_1^{\rm R}\rangle\langle\psi_1^{\rm L}|\psi_1^{\rm L}\rangle\delta(t-t') = {\rm PF}\times\frac{\theta}{2\langle\Psi|\Psi\rangle}\delta(t-t') \tag{26}$$

where the non-enhanced linewidth is $\Delta\omega_0 = \theta/(2\langle\Psi|\Psi\rangle)$ [2] and the enhanced linewidth is given by $\Delta\omega = {\rm PF}\times\Delta\omega_0$. From the above derivation, the PF enhancement is the result of two effects, the reduction of effective square amplitude ($|c_1|^2 = \langle\Psi|\Psi\rangle/\langle\psi_1^{\rm R}|\psi_1^{\rm R}\rangle$) and the enhancement of noise by $\langle\psi_1^{\rm L}|\psi_1^{\rm L}\rangle$.

Up to now we have not chosen individual normalizations for $\langle\psi_1^{\rm L}|\psi_1^{\rm L}\rangle$ and $\langle\psi_1^{\rm R}|\psi_1^{\rm R}\rangle$ as they appear together in the Petermann factor. Motivated by the fact that left and right eigenvectors can be mapped onto the same Hilbert space, we select the symmetric normalization:

$$\langle\psi_1^{\rm L}|\psi_1^{\rm L}\rangle = \langle\psi_1^{\rm R}|\psi_1^{\rm R}\rangle = \sqrt{\rm PF} \tag{27}$$

With this normalization the squared field amplitude is reduced and the noise input is increased both by a factor of $\sqrt{\rm PF}$, resulting in the linewidth enhancement by a factor of PF. We note that other interpretations are possible through different normalizations. For example, in Siegman's analysis $\langle\psi_1^{\rm L}|\psi_1^{\rm L}\rangle = {\rm PF}$ and $\langle\psi_1^{\rm R}|\psi_1^{\rm R}\rangle = 1$ is chosen, and the enhancement is fully attributed to noise increase by a factor of PF [1] .

Supplementary Note 4. LANGEVIN FORMALISM

Here we analyze the system with a Langevin formalism, which includes Brillioun gain, the Sagnac effect and the Kerr effect. An Adler-like equation will be derived that provides an improved laser linewidth formula and an expression for the locking bandwidth dependence on the field amplitude ratio.

First we summarize symbols and give their definitions. For readability, all cw subscript will be replaced by $\overline{1}$ and all ccw subscript will be replaced by $\overline{2}$. The modes are pumped at angular frequencies $\omega_{{\rm P},\overline{1}}$ and $\omega_{{\rm P},\overline{2}}$. These frequencies will generally be different from the unpumped resonator mode frequency. The cw and ccw stimulated Brillouin lasers (SBLs) oscillate on the same longitudinal mode with frequency ω. This frequency is shifted for both cw and ccw waves by the same amount as a result of the pump-induced Kerr shift. On the other hand, the Kerr effect causes cross-phase and self-phase modulation of the cw and ccw waves that induces different frequency shifts in these waves. This shift and the rotation-induced Sagnac shift are accounted for using offset frequencies $\delta\omega_{\overline{1}} = -\eta\left(a_{\overline{1}}^\dagger a_{\overline{1}} + 2a_{\overline{2}}^\dagger a_{\overline{2}}\right) - \Omega\omega D/(2n_{\rm g}c)$ and $\delta\omega_{\overline{2}} = -\eta\left(a_{\overline{2}}^\dagger a_{\overline{2}} + 2a_{\overline{1}}^\dagger a_{\overline{1}}\right) + \Omega\omega D/(2n_{\rm g}c)$ relative to ω, where $\eta = n_2\hbar\omega^2 c/(Vn_0^2)$ is the single-photon nonlinear angular frequency shift, n_2 is the nonlinear refractive index, V is the mode volume, n_0 is the linear refractive index, c is the speed of light in vacuum, Ω is the rotation rate, D is the resonator diameter and $n_{\rm g}$ is the group index. Phonon modes have angular frequencies $\Omega_{\rm phonon} = 2\omega n_0 v_{\rm s}/c$ where $v_{\rm s}$ is the velocity of the phonons. The loss rate of phonon modes is denoted as Γ (also known as the gain bandwidth) and the loss rate of the SBL modes are assumed equal and denoted as γ. In addition, coupling between the two SBL modes is separated as a dissipative part and conservative part, denoted as κ and χ, respectively. These rates will be assumed to satisfy $\Gamma \gg \gamma \gg |\kappa|, |\chi|$ to simplify the calculations, which is *a posteriori* verified in our system. In the following analysis, we will treat the SBL modes and phonon modes quantum mechanically and define $a_{\overline{1}}$ ($a_{\overline{2}}$) and $b_{\overline{1}}$ ($b_{\overline{2}}$) as the lowering operators of the cw (ccw) components of the SBL and phonon modes, respectively. Meanwhile, pump modes are treated as a noise-free classical fields $A_{\overline{1}}$ and $A_{\overline{2}}$ (photon-number-normalized amplitudes).

Using these definitions, the full equations of motion for the SBL and phonon modes read

$$\dot{a}_{\overline{1}} = -\left(\frac{\gamma}{2} + i\omega + i\delta\omega_{\overline{1}}\right)a_{\overline{1}} + (\kappa + i\chi)a_{\overline{2}} - ig_{ab}A_{\overline{2}}b_{\overline{2}}^\dagger\exp(-i\omega_{{\rm P},\overline{2}}t) + F_{\overline{1}}(t) \tag{28}$$

$$\dot{a}_{\overline{2}} = -\left(\frac{\gamma}{2} + i\omega + i\delta\omega_{\overline{2}}\right)a_{\overline{2}} + (\kappa^* + i\chi^*)a_{\overline{1}} - ig_{ab}A_{\overline{1}}b_{\overline{1}}^\dagger\exp(-i\omega_{{\rm P},\overline{1}}t) + F_{\overline{2}}(t) \tag{29}$$

$$\dot{b}_{\overline{1}}^\dagger = -\left(\frac{\Gamma}{2} - i\Omega_{\rm phonon}\right)b_{\overline{1}}^\dagger + ig_{ab}A_{\overline{1}}^* a_{\overline{2}}\exp(i\omega_{{\rm P},\overline{1}}t) + f_{\overline{1}}^\dagger(t) \tag{30}$$

$$\dot{b}_{\overline{2}}^\dagger = -\left(\frac{\Gamma}{2} - i\Omega_{\rm phonon}\right)b_{\overline{2}}^\dagger + ig_{ab}A_{\overline{2}}^* a_{\overline{1}}\exp(i\omega_{{\rm P},\overline{2}}t) + f_{\overline{2}}^\dagger(t) \tag{31}$$

where g_{ab} is the single-particle Brillouin coupling coefficient. The fluctuation operators $F(t)$ and $f(t)$ associated with the field operators have the following correlations:

$$\langle F_{\overline{1}}^\dagger(t)F_{\overline{1}}(t')\rangle = \langle F_{\overline{2}}^\dagger(t)F_{\overline{2}}(t')\rangle = \gamma N_{\rm th}\delta(t-t') \tag{32}$$

$$\langle F_{\overline{1}}(t)F_{\overline{1}}^\dagger(t')\rangle = \langle F_{\overline{2}}(t)F_{\overline{2}}^\dagger(t')\rangle = \gamma(N_{\rm th}+1)\delta(t-t') \tag{33}$$

$$\langle f_{\overline{1}}^\dagger(t)f_{\overline{1}}(t')\rangle = \langle f_{\overline{2}}^\dagger(t)f_{\overline{2}}(t')\rangle = \Gamma n_{\rm th}\delta(t-t') \tag{34}$$

$$\langle f_{\overline{1}}(t)f_{\overline{1}}^\dagger(t')\rangle = \langle f_{\overline{2}}(t)f_{\overline{2}}^\dagger(t')\rangle = \Gamma(n_{\rm th}+1)\delta(t-t') \tag{35}$$

where $N_{\rm th}$ and $n_{\rm th}$ are the thermal occupation numbers of the SBL state and phonon state. In addition, there are

non-zero cross-correlations of the photon fluctuation operators due to the dissipative coupling:

$$\langle F^\dagger_{\bar{2}}(t)F_{\bar{1}}(t')\rangle = -2\kappa N_{\mathrm{th}}\delta(t-t') \tag{36}$$

$$\langle F^\dagger_{\bar{1}}(t)F_{\bar{2}}(t')\rangle = -2\kappa^* N_{\mathrm{th}}\delta(t-t') \tag{37}$$

$$\langle F_{\bar{2}}(t)F^\dagger_{\bar{1}}(t')\rangle = -2\kappa^* (N_{\mathrm{th}}+1)\delta(t-t') \tag{38}$$

$$\langle F_{\bar{1}}(t)F^\dagger_{\bar{2}}(t')\rangle = -2\kappa (N_{\mathrm{th}}+1)\delta(t-t') \tag{39}$$

All other cross correlations not explicitly written are 0.

4.1. Single SBL

We first study a single laser mode and its corresponding phonon field ($a_{\bar{1}}$ and $b_{\bar{2}}$) by neglecting κ and χ. By introducing the slow varying envelope with $a_{\bar{1}} = \alpha_{\bar{1}}\mathrm{e}^{-i\omega t}$ and $b_{\bar{2}} = \beta_{\bar{2}}\mathrm{e}^{-i(\omega_{\mathrm{P},\bar{2}}-\omega)t}$, the following equations result:

$$\dot{\alpha}_{\bar{1}} = -\left(\frac{\gamma}{2} + i\delta\omega_{\bar{1}}\right)\alpha_{\bar{1}} - ig_{ab}A_{\bar{2}}\beta^\dagger_{\bar{2}} + F_{\bar{1}}(t)\mathrm{e}^{i\omega t} \tag{40}$$

$$\dot{\beta}^\dagger_{\bar{2}} = -\left(\frac{\Gamma}{2} + i\Delta\Omega_{\bar{2}}\right)\beta^\dagger_{\bar{2}} + ig_{ab}A^*_{\bar{2}}\alpha_{\bar{1}} + f^\dagger_{\bar{2}}(t)\mathrm{e}^{-i(\omega_{\mathrm{P},\bar{2}}-\omega)t} \tag{41}$$

where we have defined the frequency mismatch $\Delta\Omega_{\bar{2}} = \omega_{\mathrm{P},\bar{2}} - \omega - \Omega_{\mathrm{phonon}}$. Neglecing the weak Kerr effect term in $\delta\omega_{\bar{1}}$ this is a set of linear equations in $a_{\bar{1}}$ and $b_{\bar{2}}$. The eigenvalues of the coefficient matrix

$$\begin{pmatrix} -\gamma/2 - i\delta\omega_{\bar{1}} & -ig_{ab}A_{\bar{2}} \\ ig_{ab}A^*_{\bar{2}} & -\Gamma/2 - i\Delta\Omega_{\bar{2}} \end{pmatrix} \tag{42}$$

can be solved as

$$\mu_{1,2} = \frac{1}{4}\left(-\Gamma - \gamma - 2i\delta\omega_{\bar{1}} - 2i\Delta\Omega_{\bar{2}} \pm \sqrt{16g^2_{ab}|A_{\bar{2}}|^2 + (\Gamma - \gamma - 2i\delta\omega_{\bar{1}} + 2i\Delta\Omega_{\bar{2}})^2}\right) \tag{43}$$

At the lasing threshold, the first eigenvalue μ_1 has a real part of 0. This can be rewritten as

$$16g^2_{ab}|A_{\bar{2}}|^2 + (\Gamma - \gamma - 2i\delta\omega_{\bar{1}} + 2i\Delta\Omega_{\bar{2}})^2 = (\Gamma + \gamma + 2i\delta\omega_{\bar{1}} + 2i\Delta\Omega_{\bar{2}} + 4i\mathrm{Im}(\mu_1))^2 \tag{44}$$

Solving this complex equation gives the SBL eigenfrequency as well as the lasing threshold,

$$\mu_1 = -i\frac{\gamma\Delta\Omega_{\bar{2}} + \Gamma\delta\omega_{\bar{1}}}{\Gamma + \gamma} \tag{45}$$

$$g^2_{ab}|A_{\bar{2}}|^2 = \frac{\Gamma\gamma}{4}\left(1 + \frac{4(\Delta\Omega_{\bar{2}} - \delta\omega_{\bar{1}})^2}{(\Gamma + \gamma)^2}\right) \tag{46}$$

The threshold at perfect phase matching ($\Delta\Omega_{\bar{2}} = \delta\omega_{\bar{1}}$) is usually written in a more familiar form $g_0|A_{\bar{2}}|^2 = \gamma/2$, where g_0 is the Brillouin gain factor [3]. Comparison gives

$$g_{ab} = \sqrt{\frac{g_0\Gamma}{2}} \tag{47}$$

We also introduce the modal Brillioun gain function for a single direction:

$$g_{\bar{1}} \equiv \frac{g_0}{1 + 4(\delta\omega_{\bar{1}} - \Delta\Omega_{\bar{2}})^2/(\Gamma + \gamma)^2} \tag{48}$$

so that the threshold can be written as

$$g_{\bar{1}}|A_{\bar{2}}|^2 = \frac{\gamma}{2} \tag{49}$$

With the threshold condition solved, the matrix can be decomposed using the bi-orthogonal approach outlined in Supplementary Note 1. The linear combination that describes the composite SBL mode can be found as

$$\overline{\alpha}_{\bar{1}} = \frac{\Gamma}{\gamma+\Gamma}\left[\alpha_{\bar{1}} - i\frac{g_{ab}}{\Gamma}\frac{2}{1+2i(\Delta\Omega_{\bar{2}}-\delta\omega_{\bar{1}})/(\Gamma+\gamma)}A_{\bar{2}}\beta_{\bar{2}}^{\dagger}\right] \tag{50}$$

where the factor $\Gamma/(\gamma+\Gamma)$ properly normalizes $\overline{\alpha}_{\bar{1}}$ so that $\overline{\alpha}_{\bar{1}} = \alpha_{\bar{1}}$ when only the SBL mode is present in the system, and we have dropped its dependence on the phase mismatch $\Delta\Omega_{\bar{2}} - \delta\omega_{\bar{1}}$ for simplicity. The associated equation of motion is

$$\frac{d}{dt}\overline{\alpha}_{\bar{1}} = -i\frac{\gamma\Delta\Omega_{\bar{2}} + \Gamma\delta\omega_{\bar{1}}}{\Gamma+\gamma}\overline{\alpha}_{\bar{1}} + \overline{F}_{\bar{1}}(t) \tag{51}$$

where the frequency term now includes a mode-pulling contribution so that the SBL laser frequency is given by,

$$\omega_{\mathrm{S},\bar{1}} = \omega + \frac{\gamma\Delta\Omega_{\bar{2}} + \Gamma\delta\omega_{\bar{1}}}{\Gamma+\gamma} \tag{52}$$

Also, we have defined a combined fluctuation operator for $\overline{\alpha}_{\bar{1}}$,

$$\overline{F}_{\bar{1}}(t) = \frac{\Gamma}{\gamma+\Gamma}\left[F_{\bar{1}}(t)\mathrm{e}^{i\omega t} - i\sqrt{\frac{1-2i(\Delta\Omega_{\bar{2}}-\delta\omega_{\bar{1}})/(\Gamma+\gamma)}{1+2i(\Delta\Omega_{\bar{2}}-\delta\omega_{\bar{1}})/(\Gamma+\gamma)}}\sqrt{\frac{\gamma}{\Gamma}}f_{\bar{2}}^{\dagger}(t)\mathrm{e}^{-i(\omega_{\mathrm{P},\bar{2}}-\omega)t}\right] \tag{53}$$

with the following correlations,

$$\langle\overline{F}_{\bar{1}}^{\dagger}(t)\overline{F}_{\bar{1}}(t')\rangle = \left(\frac{\Gamma}{\gamma+\Gamma}\right)^2\left(\langle F_{\bar{1}}^{\dagger}(t)F_{\bar{1}}(t')\rangle + \frac{\gamma}{\Gamma}\langle f_{\bar{1}}^{\dagger}(t)f_{\bar{1}}(t')\rangle\right) = \left(\frac{\Gamma}{\gamma+\Gamma}\right)^2\gamma(n_{\mathrm{th}}+N_{\mathrm{th}})\delta(t-t') \tag{54}$$

$$\langle\overline{F}_{\bar{1}}(t)\overline{F}_{\bar{1}}^{\dagger}(t')\rangle = \left(\frac{\Gamma}{\gamma+\Gamma}\right)^2\left(\langle F_{\bar{1}}(t)F_{\bar{1}}^{\dagger}(t')\rangle + \frac{\gamma}{\Gamma}\langle f_{\bar{1}}(t)f_{\bar{1}}^{\dagger}(t')\rangle\right) = \left(\frac{\Gamma}{\gamma+\Gamma}\right)^2\gamma(n_{\mathrm{th}}+N_{\mathrm{th}}+2)\delta(t-t') \tag{55}$$

We can now write $\overline{\alpha}_{\bar{1}}(t) = \sqrt{N_{\bar{1}}}\exp(-i\phi_{\bar{1}})$, where $N_{\bar{1}}$ is the photon number, $\phi_{\bar{1}}$ is the phase for the SBL, and where amplitude fluctuations have been ignored on account of quenching of these fluctuations above laser threshold. We note that amplitude fluctuations may result in linewidth corrections similar to the Henry α factor, but we will ignore these effects here. The full equation of motion for $\phi_{\bar{1}}$ is

$$\dot{\phi}_{\bar{1}} = \omega_{\mathrm{S},\bar{1}} - \omega + \Phi_{\bar{1}}(t), \quad \Phi_{\bar{1}}(t) = \frac{i}{2\sqrt{N_{\bar{1}}}}(\overline{F}_{\bar{1}}(t)\mathrm{e}^{i\phi_{\bar{1}}} - \overline{F}_{\bar{1}}^{\dagger}(t)\mathrm{e}^{-i\phi_{\bar{1}}}) \tag{56}$$

The correlation of the noise operator is given by,

$$\langle\Phi_{\bar{1}}(t)\Phi_{\bar{1}}(t')\rangle = \frac{1}{4N_{\bar{1}}}(\langle\overline{F}_{\bar{1}}^{\dagger}(t)\overline{F}_{\bar{1}}(t')\rangle + \langle\overline{F}_{\bar{1}}(t)\overline{F}_{\bar{1}}^{\dagger}(t')\rangle = \left(\frac{\Gamma}{\gamma+\Gamma}\right)^2\frac{\gamma}{2N_{\bar{1}}}(n_{\mathrm{th}}+N_{\mathrm{th}}+1)\delta(t-t') \tag{57}$$

and we identify the coefficient before the delta function,

$$\Delta\omega_{\mathrm{FWHM},\bar{1}} = \left(\frac{\Gamma}{\gamma+\Gamma}\right)^2\frac{\gamma}{2N_{\bar{1}}}(n_{\mathrm{th}}+N_{\mathrm{th}}+1) \tag{58}$$

as the full-width half-maximum (FWHM) linewidth of the SBL.

In the experiment, the frequency noise of the SBL beating signal is measured. To compare against the experiment, we calculate the FWHM linewidth for the beating signal by adding together the linewidths in two directions:

$$\Delta\omega_{\mathrm{FWHM}} = \Delta\omega_{\mathrm{FWHM},\bar{1}} + \Delta\omega_{\mathrm{FWHM},\bar{2}} = \left(\frac{\Gamma}{\gamma+\Gamma}\right)^2\left(\frac{1}{2N_{\bar{1}}}+\frac{1}{2N_{\bar{2}}}\right)\gamma(n_{\mathrm{th}}+N_{\mathrm{th}}+1) \tag{59}$$

and then convert to the one-sided power spectral density S_ν:

$$S_\nu = \frac{1}{\pi}\frac{\Delta\omega_{\mathrm{FWHM}}}{2\pi} = \left(\frac{\Gamma}{\gamma+\Gamma}\right)^2\frac{\hbar\omega^3}{4\pi^2 Q_{\mathrm{T}}Q_{\mathrm{ex}}}(\frac{1}{P_{\mathrm{cw}}}+\frac{1}{P_{\mathrm{ccw}}})(n_{\mathrm{th}}+N_{\mathrm{th}}+1) \tag{60}$$

where Q_{T} and Q_{ex} are the loaded and coupling Q factors, and P_{cw} and P_{ccw} are the SBL powers in each direction.

4.2. Two SBLs

Now we can apply a similar procedure to the two pairs of photon and phonon modes with coupling on the optical modes. We write the equations of motion for the SBL modes:

$$\frac{d}{dt}\overline{\alpha}_{\overline{1}} = -i(\omega_{\mathrm{S},\overline{1}} - \omega)\overline{\alpha}_{\overline{1}} + \frac{\Gamma}{\gamma+\Gamma}(\kappa + i\chi)\alpha_{\overline{2}} + \overline{F}_{\overline{1}}(t) \tag{61}$$

$$\frac{d}{dt}\overline{\alpha}_{\overline{2}} = -i(\omega_{\mathrm{S},\overline{2}} - \omega)\overline{\alpha}_{\overline{2}} + \frac{\Gamma}{\gamma+\Gamma}(\kappa^* + i\chi^*)\alpha_{\overline{1}} + \overline{F}_{\overline{2}}(t) \tag{62}$$

where quantities with the opposite subscript are defined similarly. We note that the coupling term involves the optical modes $\alpha_{\overline{1}}$ and $\alpha_{\overline{2}}$ only. However, no additional coupling occurs between the other components of the SBL eigenstates $\overline{\alpha}_{\overline{1}}$ and $\overline{\alpha}_{\overline{2}}$, and these states do not change up to first order of κ/γ and χ/γ. Thus we can approximate the optical mode $\alpha_{\overline{1}}$ with the composite SBL mode $\overline{\alpha}_{\overline{1}}$. Within these approximations the lasing thresholds are also the same as the independent case [4]. The equations now become

$$\frac{d}{dt}\overline{\alpha}_{\overline{1}} = -i(\omega_{\mathrm{S},\overline{1}} - \omega)\overline{\alpha}_{\overline{1}} + (\overline{\kappa} + i\overline{\chi})\overline{\alpha}_{\overline{2}} + \overline{F}_{\overline{1}}(t) \tag{63}$$

$$\frac{d}{dt}\overline{\alpha}_{\overline{2}} = -i(\omega_{\mathrm{S},\overline{2}} - \omega)\overline{\alpha}_{\overline{2}} + (\overline{\kappa}^* + i\overline{\chi}^*)\overline{\alpha}_{\overline{1}} + \overline{F}_{\overline{2}}(t) \tag{64}$$

where we have defined mode-pulled coupling rates $\overline{\kappa} = \kappa\Gamma/(\gamma+\Gamma)$ and $\overline{\chi} = \chi\Gamma/(\gamma+\Gamma)$.

We can write $\overline{\alpha}_j(t) = \sqrt{N_j}\exp(-i\phi_j)$ with $j = \overline{1}, \overline{2}$, and once again ignore amplitude fluctuations. The equations of motion for the phases are,

$$\frac{d}{dt}\phi_{\overline{1}} = (\omega_{\mathrm{S},\overline{1}} - \omega) - q\mathrm{Im}[(\overline{\kappa} + i\overline{\chi})\mathrm{e}^{(i\phi_{\overline{1}} - i\phi_{\overline{2}})}] + \frac{i}{2\sqrt{N_{\overline{1}}}}(\overline{F}_{\overline{1}}(t)\mathrm{e}^{i\phi_{\overline{1}}} - \overline{F}^{\dagger}_{\overline{1}}(t)\mathrm{e}^{-i\phi_{\overline{1}}}) \tag{65}$$

$$\frac{d}{dt}\phi_{\overline{2}} = (\omega_{\mathrm{S},\overline{2}} - \omega) - q^{-1}\mathrm{Im}[(\overline{\kappa}^* + i\overline{\chi}^*)\mathrm{e}^{(i\phi_{\overline{2}} - i\phi_{\overline{1}})}] + \frac{i}{2\sqrt{N_{\overline{2}}}}(\overline{F}_{\overline{2}}(t)\mathrm{e}^{i\phi_{\overline{2}}} - \overline{F}^{\dagger}_{\overline{2}}(t)\mathrm{e}^{-i\phi_{\overline{2}}}) \tag{66}$$

where we have defined the amplitude ratio $q = \sqrt{N_{\overline{2}}/N_{\overline{1}}}$ for simplicity. As we measure the beatnote frequency, it is convenient to define $\phi \equiv \phi_{\overline{2}} - \phi_{\overline{1}}$ from which we obtain,

$$\frac{d\phi}{dt} = (\omega_{\mathrm{S},\overline{2}} - \omega_{\mathrm{S},\overline{1}}) + \mathrm{Im}\left\{\left[q(\overline{\kappa} + i\overline{\chi}) + q^{-1}(\overline{\kappa} - i\overline{\chi})\right]\mathrm{e}^{-i\phi}\right\} + \Phi(t) \tag{67}$$

where the combined noise term and its correlation are given by

$$\Phi = -\frac{i}{2\sqrt{N_{\overline{1}}}}(\overline{F}_{\overline{1}}(t)\mathrm{e}^{i\phi_{\overline{1}}} - \overline{F}^{\dagger}_{\overline{1}}(t)\mathrm{e}^{-i\phi_{\overline{1}}}) + \frac{i}{2\sqrt{N_{\overline{2}}}}(\overline{F}_{\overline{2}}(t)\mathrm{e}^{i\phi_{\overline{2}}} - \overline{F}^{\dagger}_{\overline{2}}(t)\mathrm{e}^{-i\phi_{\overline{2}}}) \tag{68}$$

$$\langle\Phi(t)\Phi(t')\rangle = \left(\frac{\Gamma}{\gamma+\Gamma}\right)^2\left[\left(\frac{1}{2N_{\overline{1}}} + \frac{1}{2N_{\overline{2}}}\right)\gamma(N_{\mathrm{th}} + n_{\mathrm{th}} + 1) + \frac{2}{\sqrt{N_{\overline{1}}N_{\overline{2}}}}\left(N_{\mathrm{th}} + \frac{1}{2}\right)\mathrm{Re}(\kappa\mathrm{e}^{-i\phi(t)})\right]\delta(t-t') \tag{69}$$

Since both N_{th} and κ/γ is small, we will discard the last time-varying term and write

$$\langle\Phi(t)\Phi(t')\rangle \approx \Delta\omega_{\mathrm{FWHM}}\delta(t-t') \tag{70}$$

where $\Delta\omega_{\mathrm{FWHM}} = \Gamma^2/(\gamma+\Gamma)^2[(2N_{\overline{1}})^{-1} + (2N_{\overline{2}})^{-1}]\gamma(N_{\mathrm{th}} + n_{\mathrm{th}} + 1)$ is the linewidth of the beating signal far from the EP (see also the single SBL discussion).

This equation can be further simplified by introducing an overall phase shift with $\overline{\phi} = \phi - \phi_0$, where $\phi_0 = \mathrm{Arg}\left[q(\overline{\kappa} + i\overline{\chi}) + q^{-1}(\overline{\kappa} - i\overline{\chi})\right]$ and $\mathrm{Arg}(z)$ is the phase of z:

$$\frac{d\overline{\phi}}{dt} = \Delta\omega_{\mathrm{D}} - \Delta\omega_{\mathrm{EP}}\sin\overline{\phi} + \Phi(t) \tag{71}$$

with

$$\Delta\omega_{\mathrm{D}} = \omega_{\mathrm{S},\overline{2}} - \omega_{\mathrm{S},\overline{1}} = \frac{\gamma}{\Gamma+\gamma}(\omega_{\mathrm{P},\overline{1}} - \omega_{\mathrm{P},\overline{2}}) + \frac{\Gamma}{\Gamma+\gamma}\left[\eta(N_{\overline{2}} - N_{\overline{1}}) + \frac{\omega D}{n_{\mathrm{g}}c}\Omega\right] \tag{72}$$

$$\Delta\omega_{\rm EP}^2=\left|q(\overline{\kappa}+i\overline{\chi})+q^{-1}(\overline{\kappa}-i\overline{\chi})\right|^2=\left(\frac{\Gamma}{\gamma+\Gamma}\right)^2\left[\left(q+\frac{1}{q}\right)^2|\kappa|^2+\left(q-\frac{1}{q}\right)^2|\chi|^2+2\left(q^2-\frac{1}{q^2}\right)\mathrm{Im}(\kappa\chi^*)\right] \tag{73}$$

This is an Adler equation with a noisy input. It shows the dependence of locking bandwidth on the amplitude ratio and coupling coefficients. Moreover, it is clear that in the absence of $\Delta\omega_{\rm EP}$, the beating linewidth would be given by $\Delta\omega_{\rm FWHM}$. The locking term $\Delta\omega_{\rm EP}\sin\overline{\phi}$ makes the rate of phase change nonuniform and increases the linewidth.

The following part of analysis is dedicated to obtain the linewidth from this stochastic Adler equation. We define $z_\phi=\exp(-i\overline{\phi})$ and rewrite

$$\frac{d}{dt}z_\phi=-iz_\phi(\Delta\omega_{\rm D}+\Delta\omega_{\rm EP}\frac{z_\phi-z_\phi^{-1}}{2i}+\Phi) \tag{74}$$

The solution to the Adler equation is periodic when no noise is present. To see this explicitly we use a linear fractional transform:

$$z_t=\frac{(\Delta\omega_{\rm D}-\Delta\omega_{\rm S})z_\phi+i\Delta\omega_{\rm EP}}{\Delta\omega_{\rm EP}z_\phi+i(\Delta\omega_{\rm D}-\Delta\omega_{\rm S})},\quad z_\phi=-i\frac{(\Delta\omega_{\rm D}-\Delta\omega_{\rm S})z_t-\Delta\omega_{\rm EP}}{\Delta\omega_{\rm EP}z_t-(\Delta\omega_{\rm D}-\Delta\omega_{\rm S})},\quad |z_\phi|=|z_t|=1 \tag{75}$$

$$\frac{1}{z_t}\frac{d}{dt}z_t=i\Delta\omega_{\rm S}-i\frac{\Delta\omega_{\rm EP}(z_t+z_t^{-1})/2-\Delta\omega_{\rm D}}{\Delta\omega_{\rm S}}\Phi \tag{76}$$

where we introduced $\Delta\omega_{\rm S}=\sqrt{\Delta\omega_{\rm D}^2-\Delta\omega_{\rm EP}^2}$ (which has the same meaning in the main text). The noiseless solution of z_t would be $z_t=\exp(i\Delta\omega_{\rm S}t)$, and z_ϕ can be expanded in z_t as

$$z_\phi=-i\frac{\Delta\omega_{\rm D}-\Delta\omega_{\rm S}}{\Delta\omega_{\rm EP}}+2i\frac{\Delta\omega_{\rm S}}{\Delta\omega_{\rm EP}}\sum_{p=1}^{\infty}\left(\frac{\Delta\omega_{\rm D}-\Delta\omega_{\rm S}}{\Delta\omega_{\rm EP}z_t}\right)^p \tag{77}$$

where we have assumed $\Delta\omega_{\rm D}>\Delta\omega_{\rm EP}$ for convenience so that convergence can be guaranteed (For the case $\Delta\omega_{\rm D}<-\Delta\omega_{\rm EP}$ we can expand near $z_t=0$ instead of $z_t=\infty$). Thus the signal consists of harmonics oscillating at frequency $p\Delta\omega_{\rm S}$ with exponentially decreasing amplitudes. The noise added only changes the phase of z_t (as the coefficient is purely imaginary) and to the lowest order the only effect of noise is to broaden each harmonic.

The linewidth can be found from the spectral density, which is given by the Fourier transform of the correlation function:

$$W_E(\omega)\propto\mathcal{F}_\tau\{\langle z_\phi^*(t)z_\phi(t+\tau)\rangle\}(\omega) \tag{78}$$

and the correlation is given by

$$\langle z_\phi^*(t)z_\phi(t+\tau)\rangle=\left(\frac{\Delta\omega_{\rm D}-\Delta\omega_{\rm S}}{\Delta\omega_{\rm EP}}\right)^2+4\frac{\Delta\omega_{\rm S}^2}{\Delta\omega_{\rm EP}^2}\sum_{p=1}^{\infty}\left(\frac{\Delta\omega_{\rm D}-\Delta\omega_{\rm S}}{\Delta\omega_{\rm EP}}\right)^{2p}\langle z_t(t)^pz_t(t+\tau)^{-p}\rangle \tag{79}$$

where we have discarded the $\langle z_t(t)^pz_t(t+\tau)^{-q}\rangle$ $(p\neq q)$ terms since they vanish at the lowest order of $\Delta\omega_{\rm FWHM}$.

To further calculate each $\langle z_t(t)^pz_t(t+\tau)^{-p}\rangle\equiv C_p(\tau)$ we require the integral form of the Fokker-Planck equation: if $dX(t)=\mu(X,t)dt+\sigma(X,t)dW$ is a stochastic differential equation (in the Stratonovich interpretation), where W is a Wiener process, then for $f(X)$ as a function of X, the differential equation for its average reads,

$$\frac{d}{dt}\langle f(X)\rangle=\langle(\mu+\frac{\sigma}{2}\frac{\partial\sigma}{\partial X})f'(X)\rangle+\langle\frac{1}{2}\sigma^2f''(X)\rangle \tag{80}$$

Applying the Fokker-Planck equation to $C_p(\tau)$, with the stochastic equation for z_t, gives

$$\begin{aligned}\frac{dC_p(\tau)}{d\tau}&=-p\langle i\Delta\omega_{\rm S}z_t(t)^pz_t(t+\tau)^{-p}\rangle\\&\quad+p\langle\left[\frac{\Delta\omega_{\rm FWHM}}{2\Delta\omega_{\rm S}^2}\left(\Delta\omega_{\rm EP}\frac{z_t(t+\tau)+z_t^{-1}(t+\tau)}{2}-\Delta\omega_{\rm D}\right)(\Delta\omega_{\rm EP}z_t(t+\tau)-\Delta\omega_{\rm D})\right]z_t(t)^pz_t(t+\tau)^{-p}\rangle\\&\quad-p(p+1)\langle\frac{\Delta\omega_{\rm FWHM}}{2\Delta\omega_{\rm S}^2}\left(\Delta\omega_{\rm EP}\frac{z_t(t+\tau)+z_t^{-1}(t+\tau)}{2}-\Delta\omega_{\rm D}\right)^2z_t(t)^pz_t(t+\tau)^{-p}\rangle &(81)\\&\approx\left(-ip\Delta\omega_{\rm S}-p^2\frac{\Delta\omega_{\rm D}^2+\Delta\omega_{\rm EP}^2/2}{2\Delta\omega_{\rm S}^2}\Delta\omega_{\rm FWHM}\right)C_p(\tau) &(82)\end{aligned}$$

and $C_p(0) = 1$, where again $\langle z_t(t)^p z_t(t+\tau)^{-q}\rangle$ $(p \neq q)$ terms are discarded. Thus completing the Fourier transform for each term gives the linewidth of the respective harmonics. In particular, the linewidth of the fundamental frequency can be found through

$$W_{E,1}(\omega) \propto \frac{\Delta\overline{\omega}_{\mathrm{FWHM}}}{(\omega - \Delta\omega_{\mathrm{S}})^2 + \Delta\overline{\omega}^2_{\mathrm{FWHM}}/4} \tag{83}$$

with

$$\Delta\overline{\omega}_{\mathrm{FWHM}} = \frac{\Delta\omega^2_{\mathrm{D}} + \Delta\omega^2_{\mathrm{EP}}/2}{\Delta\omega^2_{\mathrm{S}}}\Delta\omega_{\mathrm{FWHM}} = \frac{\Delta\omega^2_{\mathrm{D}} + \Delta\omega^2_{\mathrm{EP}}/2}{\Delta\omega^2_{\mathrm{D}} - \Delta\omega^2_{\mathrm{EP}}}\Delta\omega_{\mathrm{FWHM}} \tag{84}$$

We see that this result is different from the Petermann factor result, which is a theory linear in photon numbers and does not correctly take account of the saturation of the lasers and the Adler mode-locking effect.

From the expressions of $\Delta\omega_{\mathrm{D}}$ [Supplementary Eq. (72)] and $\Delta\omega_{\mathrm{EP}}$ [Supplementary Eq. (73)], the beating frequency can be expressed using the following hierarchy of equations:

$$\Delta\omega_{\mathrm{S}} = \mathrm{sgn}(\Delta\omega_{\mathrm{D}})\sqrt{\Delta\omega^2_{\mathrm{D}} - \Delta\omega^2_{\mathrm{EP}}} \tag{85}$$

$$\Delta\omega_{\mathrm{D}} = \frac{\gamma}{\Gamma+\gamma}\Delta\omega_{\mathrm{P}} + \frac{\Gamma}{\Gamma+\gamma}\Delta\omega_{\mathrm{Kerr}} \tag{86}$$

$$\Delta\omega_{\mathrm{P}} = \omega_{\mathrm{P},\bar{1}} - \omega_{\mathrm{P},\bar{2}} \tag{87}$$

$$\Delta\omega_{\mathrm{Kerr}} = \eta(N_{\bar{2}} - N_{\bar{1}}) = \frac{\eta\Delta P_{\mathrm{SBL}}}{\gamma_{\mathrm{ex}}\hbar\omega} \tag{88}$$

where sgn is the sign function and we take $\Omega = 0$ (no rotation). For the Kerr shift, $\Delta P_{\mathrm{SBL}} = P_{\mathrm{ccw}} - P_{\mathrm{cw}}$ is the output power difference of the SBLs, and γ_{ex} is the photon decay rate due to the output coupling. The center of the locking band can be found by setting $\Delta\omega_{\mathrm{D}} = 0$, which leads to $\Delta\omega_{\mathrm{P}} = -(\Gamma/\gamma)\Delta\omega_{\mathrm{Kerr}}$.

We would like to remark that the equation for locking bandwidth $\Delta\omega_{\mathrm{EP}}$ in the main text does not contain the phase-sensitive term $\mathrm{Im}(\kappa\chi^*)$. This terms leads to asymmetry of the locking band with respect to q and $1/q$ and has not been observed in the experimental data. We believe its contribution can be neglected. In other special cases, $\mathrm{Im}(\kappa\chi^*)$ disappears if there is a dominant, symmetric scatterer that determines both κ and χ (e.g. the taper coupling point), or becomes negligible if there are many small scatterers that add up incoherently (e.g. surface roughness). This term can also be absorbed into the first two terms so the locking bandwidth is rewritten using effective κ, χ and a net amplitude imbalance q_0. Thus power calibration errors in the experiment may be confused with the phase-sensitive term in the locking bandwidth.

Supplementary Note 5. TECHNICAL NOISE CONSIDERATIONS

Here we briefly consider the impact of technical noise to the readout signal. Two important noise sources are temperature drifts and imprecisely-defined pump frequencies, both of which change the phase mismatch $\Delta\Omega$. For a single SBL, the phase mismatch is transduced into the laser frequency through the mode-pulling effect [Supplementary Eq. (52) and (72)], which gives a noise transduction factor of $\gamma^2/(\Gamma+\gamma)^2$. With $\gamma/\Gamma = 0.076$ fitted from experimental data the mode-pulling effect reduces pump noise by -23 dB. The Pound-Drever-Hall locking loop in the system also suppresses noise at low offset frequencies. For counter-pumping of SBLs, the pumping source are derived from the same laser, and their frequency difference is determined by radio-frequency signals, thus the system has a strong common-mode noise rejection. Within the model described by Supplementary Eq. (72), the SBL frequency is dependent on the pump frequency difference only, and features a very high common-mode noise rejection. Other effects that are not considered in the model (i.e. drift of frequency difference between pump and SBL modes) are believed to be minor for offset frequencies above 10Hz, where the Allan deviation shows a slope of $-1/2$ corresponding to white frequency noise.

Supplementary References

[1] Siegman, A. Excess spontaneous emission in non-hermitian optical systems. I. Laser amplifiers. *Phys. Rev. A* **39**, 1253 (1989).
[2] Sargent III, M., Scully, M. O. & Lamb Jr, W. E. *Laser Physics* (Addison-Wesley Pub. Co., 1974).

[3] Li, J., Lee, H., Chen, T. & Vahala, K. J. Characterization of a high coherence, Brillouin microcavity laser on silicon. *Opt. Express* **20**, 20170–20180 (2012).

[4] Lai, Y.-H., Lu, Y.-K., Suh, M.-G., Yuan, Z. & Vahala, K. Observation of the exceptional-point-enhanced Sagnac effect. *Nature* **576**, 65–69 (2019).
